# Antibacterial and Anticandidal Activity of the Nanostructural Composite of a Spirothiazolidine-Derivative Assembled on Silver Nanoparticles

**DOI:** 10.3390/molecules29051139

**Published:** 2024-03-04

**Authors:** Odeh A. O. Alshammari, Eid. M. S. Azzam, Munirah S. Alhar, Kaseb D. Alanazi, Sara A. A. Aljuhani, Walaa I. Elsofany

**Affiliations:** 1 Department of Chemistry, College of Science, University of Ha’il, Ha’il 81451, Saudi Arabia; odeh.alshammari@uoh.edu.sa (O.A.O.A.); m.alhar@uoh.edu.sa (M.S.A.); k.alanazi@uoh.edu.sa (K.D.A.); saraaljuhani683@gmail.com (S.A.A.A.); 2 Egyptian Petroleum Research Institute, Nasr City, Cairo 11727, Egypt; 3 Photochemistry Department, Chemical Industries Research Institute, National Research Centre, 33 EL Buhouth St., Dokki, Giza 12622, Egypt

**Keywords:** ionic liquid, spirothiazolidine, nanostructure, antibacterial, anticandidal

## Abstract

Our aims in this work are the preparation of an ionic liquid based on heterocyclic compounds with Ag nanoparticles and the investigation of its application as an antibacterial and anticandidal agent. These goals were achieved through the fabrication of an ionic liquid based on Ag nanoparticles with 5-Amino-3-(4-fluorophenyl)-N-hexadecyl-7-(4-methylphenyl)-2-H spiro[cyclohexane1,2’-[1,3]thiazolo [4,5-b]pyridine]-6-carbonitrile (**P16**). The nanostructure of the prepared ionic liquid was characterized using techniques such as FTIR, ^1^HNMR, ^13^CNMR, UV, SEM, and TEM. The biological activity of the prepared compound (**P16**) and its nanocomposites with Ag nanoparticles was tested using five clinical bacteria (*Pseudomonas aeruginosa* 249; *Escherichia coli* 141; *Enterobacter* cloacae 235; *Staphylococcus epidermidis* BC 161, and methicillin-resistant *S. aureus* 217), and three Candida species (*Candida utilis* ATCC 9255; *C. tropicalis* ATCC 1362, and *C. albicans* ATCC 20402). The FTIR, ^1^HNMR, and ^13^CNMR results confirmed the chemical structure of the synthesized **P16** compound. The nanostructure of the prepared ionic liquid was determined based on data obtained from the UV, SEM, and TEM tests. The antibacterial and anticandidal results showed that the biological activity of the compound (**P16**) was enhanced after the formation of nanocomposite structures with Ag nanoparticles. Moreover, the biological activity of the compound itself (**P16**) and that of its nanocomposite structure with Ag nanoparticles was higher than that of ampicillin and amphotericin B, which were used as control drugs in this work.

## 1. Introduction

**Scheme 1:**
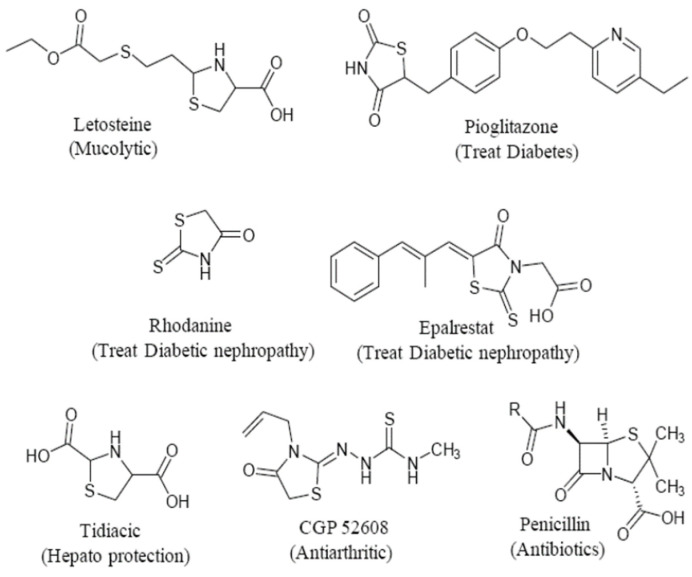
Examples of common drugs based on the thiazolidine ring.

Ionic liquids are among the most important charged surface activity agents. Recently, scientists have become interested in developing environmentally friendly, safe, and effective synthesis methods to achieve the best results in this field. Ionic liquids have been reported as green solvents and alternatives to VOCs. The characteristics of ionic liquids (ILs) are high stability, high conductivity, low vapor pressure, high temperature, and their ability to dissolve polar and nonpolar compounds [1]. They are used in organic synthesis, catalysis, food science, analytical chemistry, and nanotechnology. Also, ionic liquids are important in medicinal chemistry; due to their biological properties they are used as disinfectants, antimicrobials, and anti-inflammatory and anti-cancer agents [2]. The development, amalgamation, and production of molecules with benefits for people are among the basic objectives of therapeutic natural chemistry [3,4,5]. Recent developments in combinatorial chemistry have made it possible to obtain chemical libraries based on desirable structures [6,7,8]. A class of naturally occurring chemicals known as spiro-compounds has important biological characteristics [9,10,11]; due to the link between their structure and activity, they are a particularly engaging target for the synthesis of combinatorial libraries [12,13,14]. This group represents a sizeable class of heterocycles with nitrogen and sulfur that are commonly utilized as important building units within the pharmaceutical field [15,16,17]. Recently, an effective and additive-free green method was developed for the environmentally friendly synthesis of heterocycles with amazing yields and quick reaction times [18,19,20,21,22]. A literature survey revealed that thiazolidine is a parent compound of an array of substances that are of fundamental importance in restorative and pharmaceutical chemistry. Key medications, including pioglitazone, epalrestat, letosteine, and tidiacic, are among those that integrate the thiazolidine nucleus, which is significant in medical and pharmaceutical chemistry (Scheme 1). The nucleus of thiazolidine is also referred to as the “wonder nucleus”, since it produces a variety of derivatives with a wide range of biological functions [23,24,25]. It has been demonstrated that this compound possesses antibacterial and anti-HIV [26,27] properties due to the presence of a N-C-S linkage. Spirothiazolidines have received less attention, even though they enable conformational qualifications and original orientation of a variety of elements in spiro-compounds. This platform is recognized as a valuable isostere for known dynamic spiroimidazolidines, spirohydantoines, and spirobenzofuranes, with usage in G protein-coupled receptors (GPCRs) and peptidomimetics. It is a component of the antipsychotic medication spiclomazine. Simple spirothiazolidines have been shown to inhibit metalloprotease, giving rise to their use in the management of malaria infections [28]. Derivatives of spirothiazolidine have also been shown to have good antioxidant [29], anticancer [30], antihyperglycemic, antidiabetic [31], anti-inflammatory [32,33], anticonvulsant [34,35,36], and antimicrobial activities [37]. In this respect, nanomaterials have been receiving increased attention in recent years. Notably, silver nanomaterials show a noteworthy contrast to other macroscopic and small-molecular species due to their particle sizes. They have broad applications within the chemical industry, i.e., in medications, biological agents, superconductors, and other fields [38]. Considering these advantages, the primary goals of our work are to synthesize a biologically important scaffold based on spiro(cyclohexane-thiazolidine) derivatives and related nanocomposites and to evaluate the antibacterial and antifungal activity of these materials.

## 2. Results and Discussion

### 2.1. Confirmation of the Structure of ***P16***

**Figure 1:**
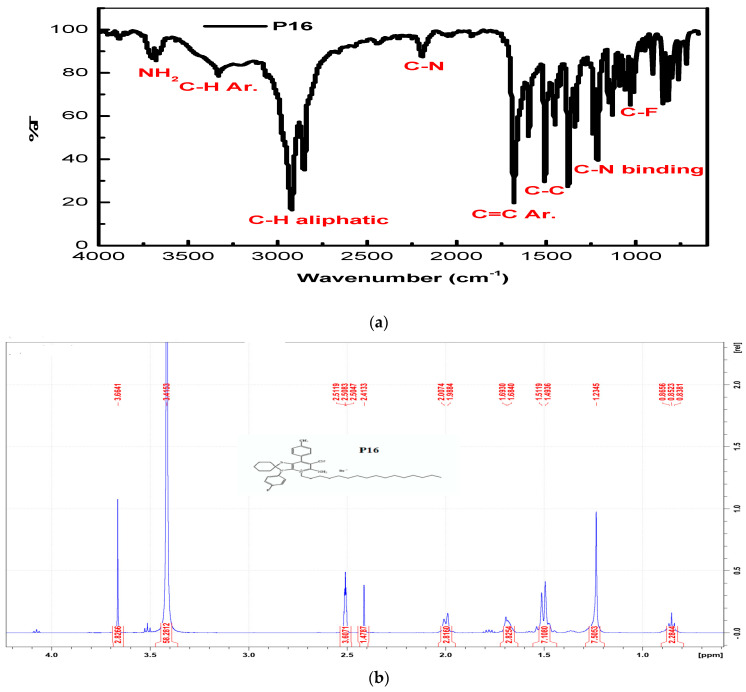

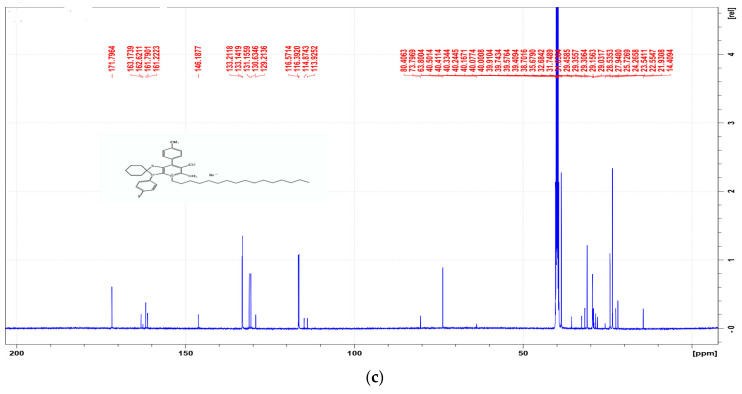
FTIR (**a**), H^1^NMR (**b**), and ^13^CNMR (**c**) spectra of compound **P16**.

The FTIR spectra of **P16**, as shown in Figure 1, reveal peaks at 870 cm^−1^ (C-F), 1540 cm^−1^ (C-C), 1620 cm^−1^ (C=C), 2221 cm^−1^ (CN), 2860 cm^−1^, 2925 cm^−1^ for asymmetric and symmetric stretching (CH aliphatic), 3332 cm^−1^ (C-H aromatic), and 3692 cm^−1^ (NH_2_). In addition, we also confirmed the structure of **P16** using ^1^H-NMR, as shown in (Figure 1). Figure 1 shows peaks at δ equal 0.865 (t, 3H, CH_2_-CH_3_), 1.23–1.68 (m, 26H, 13CH_2_), 1.69–2.007 (m, 10H, 5CH_2_ cyclohexane), 2.41 (s, 3H, Ar-CH_3_), 3.41 (m, 2H, N-CH_2_-CH_2_), 3.66 (t, 2H, CH_2_-N), 7.22–7.87 (m, 9H, Ar-H), and 8.47 ppm (broad, s, 2H, NH_2_, D_2_O exchangeable). Moreover, the structure of **P16** was investigated using ^13^C-NMR, as shown in Figure 1. The following peaks appeared at δ = 14.41 (CH_3_), 21.93 (Ar-CH_3_), 22.55–40.50 (14 CH_2_ + 5 CH_2_ cyclohexane), 63.80 (=N-CH_2_), 73.79 (c-spiro), 80.40 (CN), 113.92–133.21 (Ar-C), 146.18 (=C-N), 161.22 (C=N), 162.96 (C-NH_2_), and 163.17 ppm (C-F).

### 2.2. Nanostructure Formation of the ***P16*** with AgNPs (***P16-AgNPs***) 

**Figure 2:**
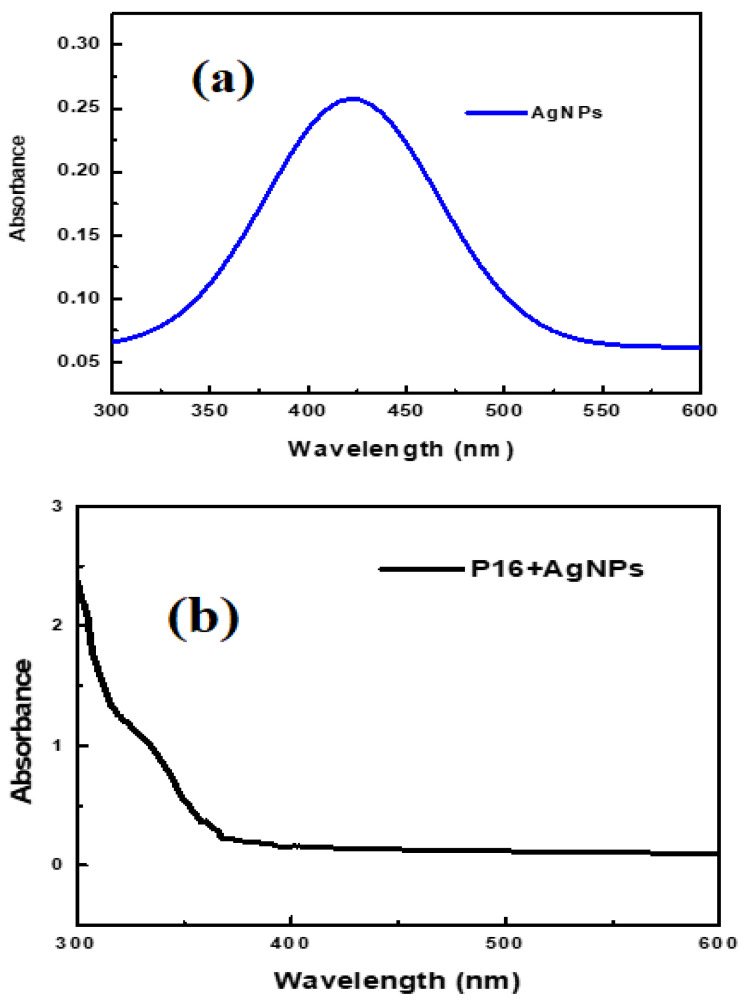
UV spectrum of AgNPs (**a**) and **P16-AgNPs** (**b**) in aqueous solution.

The first technique used in this work to establish the nanostructure of **P16** with Ag-nanoparticles was based on the UV spectrum. The UV spectrum of the prepared Ag-nanoparticles is shown in Figure 2. This figure shows the excitation and emission spectra of the Ag-nanoparticles at ~422 nm^−1^. This peak appeared because of the surface plasmon absorption of silver clusters [39]. The UV results of the nanostructure of the synthesized **P16** with the Ag-nanoparticles are represented in Figure 2. These results reveal that the peak of the AgNPs at 422 nm completely disappeared; this was due to the reduction of the negative charge that surrounded the particles as result as of the assembly of **P16** molecules on AgNPs; it is known that metal particles in aqueous colloidal dispersions usually have a negative charge due to the adsorption of anions. The addition of neutral molecules such as **P16** neutralizes the adsorbed ions and reduces the charge on these particles. In addition, the presence of **P16** on AgNPs formed nano shells instead of the nanoparticles and prevented the aggregation of AgNPs [39].

**Figure 3:**
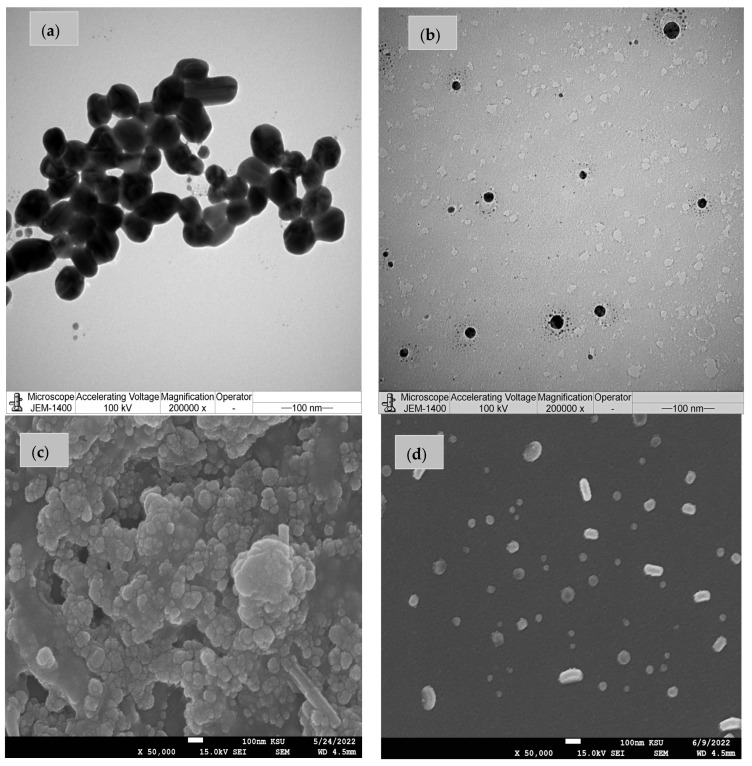
TEM images of AgNPs (**a**) and **P16-AgNPs** (**b**) and SEM images of AgNPs (**c**) and **P16-AgNPs** (**d**).

The second technique used in this work to establish the nanostructure of the **P16** with Ag-nanoparticles was TEM. Figure 3 presents TEM micrographs of the individual AgNP solution and AgNPs in the presence of **P16**. The nanostructure of the AgNPs in Figure 3 can be seen to comprise a spherical shape and polycrystalline structure, with an average size ranging from 19.1 to 21.44 nm. After capping the **P16** molecules on the AgNPs, nanoshells were formed, restricting the aggregation of AgNPs due to the formation of nanoshells with **P16** molecules (see Figure 3). In addition, the assembly of **P16** molecules increased the stability of the AgNPs and reduced their aggregation. Therefore, the presence of alkyl chains (hexadecyl) in compound **P16** enhanced the stability of the AgNPs. It was concluded that the alkyl chain length provided a physical barrier, preventing aggregation between the surfaces of AgNPs during collisions, in addition to the effect of **P16** molecules on the charge of AgNPs; that is, the **P16** reduced the surface charge of the AgNPs [40]. These factors led to an overall reduction in the aggregation of AgNPs, as shown in Figure 3. The SEM images in Figure 3 were used to further investigate the formation of the nanostructure of AgNPs and **P16-AgNPs**. It is clear from the SEM image presented in Figure 3 that the sizes of the AgNPs ranged from 43.7–51.7 nm. Moreover, the SEM image in Figure 3 gives an indication about the aggregation of the Ag-nanoparticles which were produced as a result of the surface plasmon charges on the silver nanoparticles. It is known that metal particles in colloidal solution usually have a negative charge due to adsorbed anions; this leads to more aggregation of such particles in solution, as shown in Figure 3 [40]. Further investigation of the nanostructure of the **P16** capped AgNPs (**P16-AgNPs**) is summarized in Figure 3. It was noted from previous publications that the aggregation of the nanoparticles can be weakened by mixing with neutralized molecules such as **P16**, as these neutralize the adsorbed ions and reduce the charge on the particles, thereby preventing the particles from aggregating with each other [40]. The SEM images in Figure 3 reveal the formation of nanoshells of **P16** after capping with AgNPs. This capping produced nanoshells instead of nanoparticles and prevented the aggregation of the Ag-nanoparticles, as shown in Figure 3.

### 2.3. Antibacterial Activity of ***P16*** and ***P16-AgNPs***

**Figure 4:**
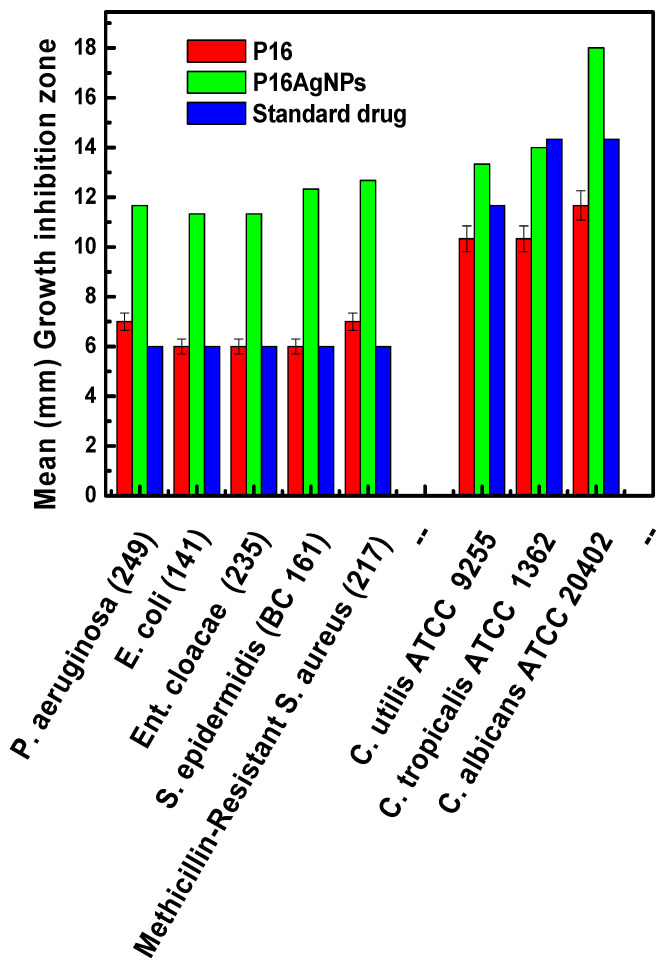
Comparison of the effect of **P16**, **P16-AgNPs,** and the control drugs (ampicillin and amphotericin B) on the growth inhibition of five clinical bacteria (*Pseudomonas aeruginosa* 249; *Escherichia coli* 141; *Enterobacter* cloacae 235; *Staphylococcus epidermidis* BC 161, and Methicillin-Resistant *S. aureus* 217) and three *Candida* species (*Candida utilis* ATCC 9255; *C. tropicalis* ATCC 1362, and *C. albicans* ATCC 20402).

**Figure 5:**
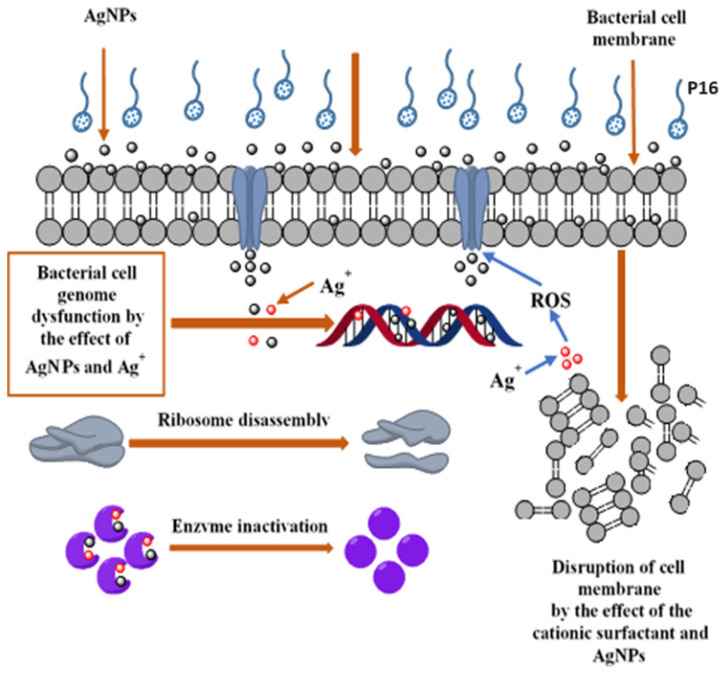
Disruption of the cell membrane and damage to cellular proteins with **P16** and **P16AgNPs**.

**P16** and its solution with AgNPs (**P16-AgNPs**) were tested to determine their abilities to inhibit the growth of micro-organisms, i.e., five clinical bacteria (*Pseudomonas aeruginosa* 249; *Escherichia coli* 141; *Enterobacter* cloacae 235; *Staphylococcus epidermidis* BC 161, and Methicillin-Resistant *S. aureus* 217) and three *Candida* species (*Candida utilis* ATCC 9255; *C. tropicalis* ATCC 1362, and *C. albicans* ATCC 20402), as shown in Table 1 and Table 2 and Figure 4. For cationic surfactants such as **P16**, it was found that the antimicrobial activity depended mainly on the aliphatic chain length; this phenomenon is known as the cut-off effect [41,42]. Many parameters are dependent upon the occurrence of the cut-off effect, including the critical micelle concentration of the surfactant, a change in the free energy of the adsorption of the cationic surfactant on the bacterial cell membrane, the hydrophobic properties, and the size of the diffused surfactant [43]. Optimal antimicrobial activity is achieved in medium chain length (C8–C18) cationic surfactants, while surfactants with alkyl chain less than C4 or more than C18 are mostly inactive [44]. Moreover, in the medium chain length range, an increase in the aliphatic chain length of the surfactant decreases the critical micelle concentration, thus lowering the surfactant concentration at the cell membrane. Accordingly, the surfactant activity will be greater with an alkyl chain length ranging from 10 to 18 carbon atoms. On the other hand, the adsorption capacity at the membrane interface should increase with increasing the hydrophobicity of the surfactant. Gram-negative bacteria are more antibiotic-resistant than Gram-(+ev) bacteria, yeast, and fungi. The outer membrane, which is composed of lipopolysaccharide molecules and periplasmic space in the Gram (-ev) bacterial cell, prevents the penetration of antibacterial agents, which make it more antibiotic-resistant [45,46,47]. Our results, as shown in Table 1 and Table 2 and Figure 4, are in clear agreement with the previously mentioned phenomena (cut-off effect) [41,42], as the activity of **P16-AgNPs** against the five clinical bacteria and the three *Candida* species under investigation was higher than that of **P16** without AgNPs. In addition, the biological activity of **P16** alone and its nanostructures with Ag-nanoparticles was higher than that of the Ampicillin or amphotericin B, which were used as control drugs. Generally, the results were good; after the formation of a nanostructure of **P16** with AgNPs, its biological activity clearly improved. As detailed in Table 1 and Table 2, the inhibition zone of **P16** increased after capping with the AgNPs. It can be seen from the results in Table 1 and Table 2 and Figure 4 that the biological activity of **P16** toward most bacteria and candida strains was high. This may be related to the fused pyridine ring with spirothiazolidine in the chemical structure of **P16**, which this likely played an important role in enhancing the biological activity [37]. Moreover, nucleophilic groups (NH_2_, CN) and the presence of electron-donating groups (CH_3_, hexadecyl) likely also resulted in the high degree of biological activity of **P16** [37]. These results indicate that **P16** favored the dispersion of AgNPs and improved their biocompatibility. The inhibitory effect of the capped AgNPs may be attributed to the generation of free radicals and Reactive Oxygen Species (ROS) in microbial cells, leading to the disruption of the cell membrane and damage to cellular proteins, as shown in Figure 5 [48].

## 3. Materials and Experimental Techniques 

### 3.1. Synthesis of 5-Amino-3-(4-fluorophenyl)-N-hexadecyl-7-(4-methylphenyl)-2-H-spiro[cyclohexane-1,2’-[1,3]thiazolo [4,5-b]pyridine]-6-carbonitrile (***P16***)

**Scheme 2:**
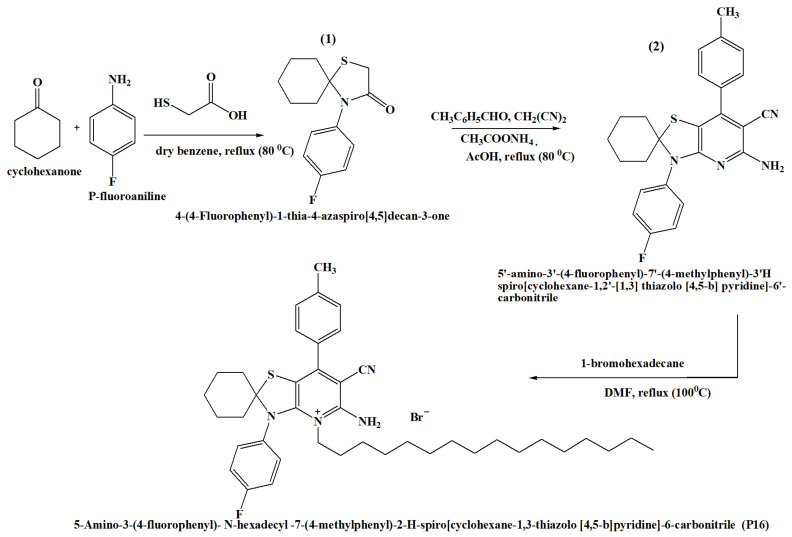
Synthesis of 5-Amino-3-(4-fluorophenyl)-N-hexadecyl-7-(4-methylphenyl)-2-H spiro[cyclohexane-1,2’-[1,3]thiazolo [4,5-b]pyridine]-6-carbonitrile (**P16**).

Compound **P16** was synthesized according to the steps shown in Scheme 2:

#### 3.1.1. Synthesis of 4-(4-Fluorophenyl)-1-thia-4-azaspiro [4,5]decan-3-one (Compound **1**)

Compound **1** was prepared by the reaction of cyclohexanone (0.01 mol), p-fluoroaniline (0.01 mol), and thioglycolic acid (0.01 mol) in dry toluene (50 mL). This mixture was then refluxed for 10 h. After filtration, drying, and recrystallization of the product from the dioxane/methanol mixture, pale-yellow needles of compound **1** were obtained [29].

#### 3.1.2. Synthesis of 5’-Amino-3’-(4-fluorophenyl)-7’-(4-methylphenyl)-3’H-spiro[cyclohexane-1,2’-[1,3]thiazolo [4,5-b]pyridine]-6’-carbonitrile (Compound **2**)

For the preparation of compound **2**, compound **1** (0.01 mol), p-tolualdehyde (0.01 mol), ammonium acetate (0.02 mol), and malononitrile (0.01 mol) in glacial acetic acid (40 mL) were refluxed for 24 h. This mixture was cooled and poured into water to obtain a solid precipitate. After filtration, drying, and recrystallization from dioxan, a deep yellow powder comprising compound **2** formed [29].

#### 3.1.3. 5-Amino-3-(4-fluorophenyl)-N-hexadecyl-7-(4-methylphenyl)-2-H-spiro[cyclohexane-1,2’-[1,3]thiazolo [4,5-b]pyridine]-6-carbonitrile (**P16**)

A solution of compound **2** (0.02 mol) in 50 mL DMF was prepared at room temperature. To this was added 1-bromhexadecane (0.02 mol). The mixture was then heated to 100 °C for 6 h. After cooling and the addition of diethyl ether, the product was precipitated. The crude precipitate was recrystallized twice from methanol to yield 5-Amino-3-(4-fluorophenyl)-N-hexadecyl-7-(4-methylphenyl)-2-H-spiro[cyclohexane-1,2’-[1,3]thiazolo [4,5-b]pyridine]-6-carbonitrile (**P16**) in the form of brown crystals (yield 80%; m.p. 131–133 °C) [40].

### 3.2. Synthesis of Silver Nanoparticles (AgNPs)

An Ag-nanoparticle colloidal solution was synthesized using the chemical reduction method shown in previous publication [40]. Briefly, 0.01 g AgNO_3_ was placed in 50 mL of distilled water the solution was boiled to 100 °C. Then, 5 mL of 0.01 g 1% sodium citrate solution was added with stirring and the solution was heated to mix the components until the color changed to pale yellow. Then, heating was stopped and the solution was continuously stirred and cooled to room temperature.

### 3.3. Nanostructure of AgNPs with Compound ***P16*** (***P16-AgNPs***)

A nanostructure of 5-Amino-3-(4-fluorophenyl)-N-hexadecyl-7-(4-methylphenyl)-2-H-spiro[cyclohexane-1,2’-[1,3] thiazolo [4,5-b]pyridine]-6-carbonitrile (**P16**) with Ag-nanoparticles was fabricated according to the following method: a solution of **P16** was prepared by dissolving it in propanol solution under continuous stirring for 1 h. Then, 20 mL of Ag-nanoparticles aqueous solution was mixed with 5 mL of the **P16** solution. After that, the mixture was stirred until the pale yellow AgNPs became colorless [40].

### 3.4. Experimental Techniques

#### 3.4.1. Confirmation of the Chemical Structure of **P16**

The FTIR spectra of the prepared **P16** was determined at the College of Science, University of Ha’il, Ha’il, KSA using a Thermo Nicolet 6700 FT-IR optical spectrometer (Mundelein, IL 60060, USA). The proton nuclear magnetic resonance (^1^HNMR) spectra of the synthesized compound (**P16**) were determined using a 850 MHz Ascend 850 Mhz NMR spectrometer (Bruker, Billerica, MA, USA). Tetramethylsilane was used as an internal reference and DMSO as a solvent. The samples were measured at the College of Science, University of King Abdulaziz, Jeddah, KSA. In addition, the chemical structure of the synthesized P16 was confirmed using ^13^CNMR spectra data. Analyses were carried out using an 850 MHz Ascend 850 Mhz NMR spectrometer (Bruker, Billerica, MA USA). Tetramethylsilane was used as an internal reference and DMSO as a solvent. Analyses were carried out at the College of Science, University of King Abdulaziz, Jeddah, KSA.

#### 3.4.2. Confirmation the Nanostructure of AgNPs with **P16** (**P16-AgNPs**)

We used UV, SEM, and TEM techniques to confirm the nanostructure of **P16** with AgNPs as follows: UV measurements were carried out with a UV–VIS double beam PC scanning spectrophotometer (LABOMED, Inc., UV-2950, Los Angeles, CA, USA). With distilled water as a solvent, the samples were tested at the College of Science, University of Ha’il, Ha’il, KSA. Scanning electron microscope (SEM) images and Energy-dispersive X-ray spectroscopy data were obtained using a SEM-EDX (JEOL JSM 7610F, Tokyo, Japan) system operating at 20 kV. All samples were tested at the College of Science, King Saudi University, Riyadh, KSA. To investigate the nanostructures of the prepared samples, TEM images (JEM-1400 Flash, Los Angeles, CA, USA) were used. The samples in our work were tested at the College of Science, University of King Saud, Riyadh, KSA.

### 3.5. Antibacterial Properties of the Synthesized ***P16*** and ***P16-AgNPs***

Both disc diffusion assay and the microdilution technique were used to assess the antibacterial activities of **P16** and **P16** with AgNPs (**P16-AgNPs**), in line with the protocol described by Snoussi et al. (2022) [49]. For the disc diffusion technique, pure colonies on bacteria cultivated on Mueller-Hinton agar medium and yeast on Sabouraud chloramphenicol agar were used to prepare a homogenous suspension. The tested microorganisms comprised five clinical bacteria (*Pseudomonas aeruginosa* 249; *Escherichia coli* 141; *Enterobacter* cloacae 235; *Staphylococcus epidermidis* BC 161, and Methicillin-Resistant *S. aureus* 217) and three *Candida* species (*Candida utilis* ATCC 9255; *C. tropicalis* ATCC 1362, and *C. albicans* ATCC 20402). A cotton swab was used to inoculate fresh Petri dishes. Sterile filter paper discs (6 mm in diameter, Biolife, Italy) were impregnated with 10 µL of both the **P16** and **P16-AgNPs** solutions (2000 ppm). Then, the discs were placed on cultured plates. The treated Petri dishes were kept for 1 h at 4 °C and then incubated at 37 °C for 24 h. We measured the growth inhibition zone (GIZ) diameter around the discs to test the antibacterial efficiency of the samples. All tests were performed in triplicate and the mean diameter of the inhibition zone was calculated. The microdilution method was used to determine the minimum inhibitory concentrations (MICs) and minimum bactericidal concentrations/minimum fungicidal concentrations (MBCs/MFCs) values of the tested compounds. For the experiment, a stock solution was prepared in 5% DMSO. Then, twofold serial dilutions of the essential oil were prepared in 96-well plates, starting from 1000 ppm/mL to 0.976 ppm/mL, in Mueller-Hinton Broth for bacteria. Next, 5 µL of the microbial inoculum was added to each well of the microtiter plate containing 0.1 mL of the serially diluted compounds. Incubation was done for 24 h at 37 °C. The minimum inhibitory concentrations (MICs) were defined as the lowest concentration of compound able to inhibit the growth of the tested microorganisms. To determine the MBCs/MFCs values, 3 µL from the wells medium with no visible growth was removed and inoculated in Mueller-Hinton agar plates for the bacterial strains and Sabouraud dextrose agar for the *Candida* strains. After 24 h of incubation at 37 °C, the growth of the microorganism was observed. The concentrations at which the microorganisms were killed (no growth) were recorded as the minimum bactericidal concentrations or the minimum fungicidal concentrations. Ampicillin (AMP; 10 μg/disc) and amphotericin B (10 mg/mL; 10 μL/disc) were used as control drugs. All biological tests were conducted in the Biology Department of the College of Science, University of Ha’il, Ha’il, KSA. 

### 3.6. Statistical Analysis

All analyses were done in triplicate and the results are shown as mean values ± SD (standard deviations). Differences in the means were calculated using Duncan’s multiple range tests for means with a 95% confidence interval (*p* < 0.05). The biological tests were conducted in the Biology Department of the College of Science, University of Ha’il, Ha’il, KSA. 

## 4. Conclusions

Ionic liquids (ILs) have attracted interest due to their antibacterial and antimicrobial activities. Recently, nanomaterials have also received considerable attention from researchers. The most important of these nanomaterials are based on silver. In the present work, we investigated the fabrication of ionic liquids based on Ag-nanoparticles with 5-Amino-3-(4-fluorophenyl)-N-hexadecyl-7-(4-methylphenyl)-2-H-spiro[cyclohexane-1,2’-[1,3]thiazolo [4,5-b]pyridine]-6-carbonitrile (**P16**). In addition, we studied the biological activity of the fabricated ionic liquids against five clinical bacteria and three *Candida* species. The overall results confirmed that the fabricated ionic liquid had high as antibacterial and antimicrobial activities. The **P16AgNPs** showed higher activity against the five clinical bacteria and the three *Candida* species than **P16** alone. Moreover, the efficiency of **P16AgNPs** was higher than that of the Ampicillin and amphotericin B, which were used as control drugs. 

## Figures and Tables

**Table 1:** Antibacterial and anti-Candida activities of **P16** and **P16-AgNPs**, as compared to those of ampicillin and amphotericin B, tested using disc diffusion assay.

**Clinical Bacterial Strains**	**P16** **Mean GIZ ± SD (mm)**	**P16-AgNPs** **Mean GIZ ± SD (mm)**	**Ampicillin** **(10 µg/disc)** **Mean GIZ ± SD (mm)**
*P. aeruginosa* (249)	7.00 ± 0.00 ^bB^	11.67 ± 0.58 ^aC^	6.00 ± 0.00 ^aA^
*E. coli* (141)	6.00 ± 0.00 ^aA^	11.33 ± 0.58 ^aB^	6.00 ± 0.00 ^aA^
*Ent. cloacae* (235)	6.00 ± 0.00 ^aA^	11.33 ± 0.58 ^aB^	6.00 ± 0.00 ^aA^
*S. epidermidis* (BC 161)	6.00 ± 0.00 ^aA^	12.33 ± 0.58 ^abB^	6.00 ± 0.00 ^aA^
Methicillin-Resistant *S. aureus* (217)	7.00 ± 0.00 ^bA^	12.67 ± 0.58 ^abB^	6.00 ± 0.00 ^aA^
**Candida Strains**	**P16** **Mean GIZ ± SD (mm)**	**P16-AgNPs** **Mean GIZ ± SD (mm)**	**Amphotericin B** **(10 mg/mL; 10 µL/disc)** **Mean ± SD (mm)**
*C. utilis* ATCC 9255	10.33 ± 0.58 ^cA^	13.33 ± 0.58 ^bcC^	11.67 ± 0.58 ^bB^
*C. tropicalis* ATCC 1362	10.33 ± 0.58 ^cA^	14.00 ± 1.00 ^cB^	14.33 ± 0.58 ^cB^
*C. albicans* ATCC 20402	11.67 ± 0.58 ^dA^	18.00 ± 1.00 ^dC^	14.33 ± 0.58 ^cB^

The inhibition zone around the discs impregnated with **P16** and **P16AgNPs** is expressed as the mean of three replicates (mm ± SD). SD: standard deviation. a–d, A–C: Each value represents an average of three repetitions. The means followed by the same letters were not significantly different at *p* = 0.05, based on Duncan’s multiple-range test. Small letters are used to compare the mGIZ of the compound, compound-AgNPs, and the control drugs with different strains, while capital letters are used to compare the mGIZ of the compound **P16**, **P16-AgNPs,** and the control drugs for the same strain.

**Table 2:** Determination of MICs and MBCs/MFCs values using microdilution assay for both **P16** and **P16-AgNPs**.

**Clinical Bacterial Strains**	**P16** **(ppm/mL)**			**P16-AgNPs** **(ppm/mL)**		
	**MIC**	**MBC**	**MBC/MIC Ratio**	**MIC**	**MBC**	**MBC/MIC Ratio**
*P. aeruginosa* (249)	62.5	1000	16; Bacteriostatic	31.25	250	8; Bacteriostatic
*E. coli* (141)	62.5	1000	16; Bacteriostatic	31.25	250	8; Bacteriostatic
*Ent. cloacae* (235)	62.5	250	4; Bactericidal	31.25	125	4; Bactericidal
*S. epidermidis* (BC 161)	31.25	250	8; Bacteriostatic	15.62	125	8; Bacteriostatic
Methicillin-Resistant *S. aureus* (217)	62.5	1000	16; Bacteriostatic	3.81	15.62	4; Bactericidal
**Candida Strains**	**P16** **(ppm/mL)**			**P16-AgNPs** **(ppm/mL)**		
	**MIC**	**MFC**	**MFC/MIC Ratio**	**MIC**	**MFC**	**MFC/MIC Ratio**
*C. utilis* ATCC 9255	250	1000	4; Fungicidal	125	250	2; Fungicidal
*C. tropicalis* ATCC 1362	250	1000	4; Fungicidal	125	250	2; Fungicidal
*C. albicans* ATCC 20402	62.5	250	4; Fungicidal	15.62	62.5	4; Fungicidal

## Data Availability

The original contributions presented in the study are included in the article material, further inquiries can be directed to the corresponding authors.
